# Study of Woad (*Isatis tinctoria* L.)-Extracted Indoxyl Precursors Conversion into Dyes: Influence of the Oxidative Media on Indigo Recovery Yields and Indigotin/Indirubin Ratio Measured by HPLC-DAD Method

**DOI:** 10.3390/molecules29204804

**Published:** 2024-10-11

**Authors:** Romain Vauquelin, Léa Juillard-Condat, Nicolas Joly, Nathalie Jullian, Elodie Choque, Patrick Martin

**Affiliations:** 1 Unité Transformations & Agroressources, Université d’Artois—UniLaSalle, ULR7519, F-62408 Béthune, France; romain.vauquelin@univ-artois.fr (R.V.); patrick.martin@univ-artois.fr (P.M.); 2 BioEcoAgro-Biologie des Plantes et Innovation, UMRT INRAe 1158 BioEcoAgro, Université de Picardie Jules Verne, F-80039 Amiens, France; nathalie.jullian@u-picardie.fr (N.J.); elodie.choque@u-picardie.fr (E.C.)

**Keywords:** *Isatis tinctoria* L., indigo, HPLC, acid hydrolysis, tetrachloroferrate

## Abstract

The production of indigo, primarily used by the denim industry, increases year by year, and is mainly of synthetic origin. The textile industry, on which its production depends, is responsible for 10% of greenhouse gases and 20% of water pollution. However, the source of this pigment/colorant, mainly based on petrochemistry, remains a key issue today. Extracting indigo from plants is becoming a popular answer and requires an understanding and evaluation of the entire process, from raw material to pigment recovery. In this study, the indigotin precursor, indoxyl, derived from the hydrolysis of *O*-glycosides biomass extracted in water, was oxidized to obtain the desired pigment. This step is the most sensitive, as variations have been observed during this phase. Consequently, the standardization of the oxidation process was established to determine the extract capacity to consistently produce the blue dye pigment. Partial hydrolysis of the *O*-glycosides, the indoxyl precursors, was identified as a factor causing this yield variability in the obtained extracts. Once the precursors were fully chemically hydrolyzed, plants harvested during summer and during a freezing period showed a similar capacity to produce indigotin, with values of 412 ± 25 ppm and 379 ± 0 ppm, respectively. This result showed that in freezing conditions, the enzymatic material was not available, resulting in the lack of indigotin formation. To address the use of oxidation in an alkaline medium, a spontaneous oxidation method was proposed. This method produced a purer indigotin pigment, with a 21.6% purity compared to 5.9% purity using air-mediated oxidation in an alkaline medium.

## 1. Introduction

Today, indigo production reaches up to 80,000 tons annually [1,2], with 45,000 tons used by the denim industry [3]. The produced pigments are mainly synthetic, whereas previously they were derived from indigo-bearing plants. Among these, woad (*Isatis tinctoria* L.) enjoyed a long historical period in Europe during the Middle Ages, particularly in France [4], England [5], and Sicily [6,7]. The production of other indigo-yielding plants, such as *Indigofera tinctoria* [8], along with the emergence of synthetic pigments [9], led to the loss of woad’s status as the sole source of indigo. The current issue is ecological. Today, the textile industry is one of the most representative sectors of the global industrial structure and ranks as the second most polluting industry in the world [10]. It is largely responsible for the consumption of non-renewable resources and contributes to nearly 10% of global greenhouse gas emissions [11]. Textile dyeing is a major source of this pollution, with 95% of dyes produced worldwide intended for this industry. Indigotin, the main dye used for denim production, accounts for around 4 billion garments each year [12]. According to the World Bank, textile dyeing industries are responsible for 20% of industrial water pollution, generating 2.1 billion tons of waste, most of which is discharged into aquatic ecosystems, primarily during the dyeing process [13].

There is an urgent need to significantly rethink indigo production as well as dyeing techniques. For dyeing, innovative and sustainable techniques appear promising, without the use of indigo or reducing agents for pigment solubilization [14]. Regarding the synthesis of indigo, recombinant processes using micro-organisms and their enzymes have also shown interesting progress [1]. However, their implementation remains complex without the necessary equipment and scientific knowledge.

A simpler and less costly alternative is to recover indigotin through infusion and the processing of extracts from woad leaves. In this context, local producers can not only extract indigo from these plants but also carry out dyeing with the obtained product. This approach could help to reduce greenhouse gas emissions within a short supply-chain dynamic, while also supporting the local economy. This approach involves the use of plants for their specialized metabolites, which offer high value-added products in various fields, such as agriculture, industry, and human health, while adhering to a circular economy dynamic [15].

The objective of this study is to enhance the value of woad in various application fields, including dyeing, through the optimization of indigo extraction from woad cauline leaves. A focus on the historical infusion process was made because it allows for the rapid production of indigo using a classical installation and an environmentally friendly non-toxic solvent, i.e., water. 

**Figure 1:**
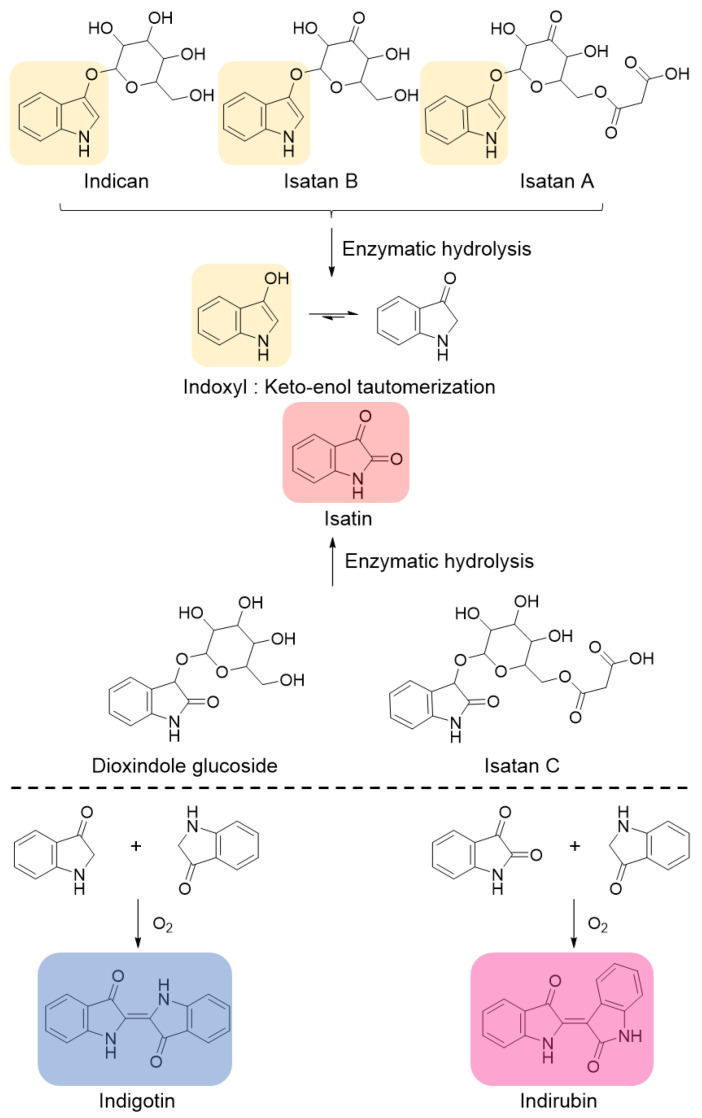
Summary diagram of the reactions of the indigo production process (adapted from [19,22,24]).

Within the vacuoles of the leaves [16] are indole alkaloid *O*-glycosides (Figure 1) that are hydrolyzed into indoxyl, which dimerizes and leads to indigotin in alkaline and aerated conditions [17,18]. In indigo-producing plants, the main glycoside responsible for the blue pigment is indoxyl-β-D-glucoside, also called indican. Three other characterized heterosides are only present in the *Isatis genus*, as follows: Isatan A; Isatan C; and Isatan B [19]. Isatan B was later revised and identified as having the most abundant *O*-glycosides in the *Isatis genus* [20]. Some of these precursors can be enzymatically hydrolyzed in situ by endogenous β-glucosidases within the leaves and during infusion [21]. Isatan C and dioxindole glucoside lead, via enzymatic hydrolysis, to the production of isatan, which is also an oxidation product of indoxyl [22]. The reaction between indoxyl and isatan produces indirubin, a ruby pigment known for its numerous biological properties, especially against leukemia [23].

In the infusion process, various parameters can present notable influences, particularly indoxyl oxidation in alkaline conditions, leading to significantly different results, even from the same woad extract. Accordingly, in order to optimize the process, it is necessary to obtain a robust, rapid, and reproducible method to oxidize indoxyl into indigotin. Reviewing studies in the literature, Caggiano and coworkers (2023) developed a simple method to indirectly measure indoxyl sulfate production by oxidizing it to indigotin using iron (III) chloride [25]. In addition, this standardization of the oxidation process also ultimately makes it possible to optimize the previous phase of the overall process and therefore maximize the extraction of indoxyl and its precursors.

In this work, an HPLC analysis methodology was developed for both indigotin and indirubin identification and quantification, allowing for the determination of the ratio obtained during oxidation processes. The results of the classical oxidation pathway (in alkaline conditions) are presented, highlighting reproducibility issues. Therefore, this oxidation was compared with other methods especially using iron (III) chloride in an acidic medium. This acidic medium oxidation revealed a partial hydrolysis of precursors from the extraction batch of summer leaves and no hydrolysis from the extraction batch of freezing period leaves. A method for indigotin production through spontaneous oxidation without additional reagents is also presented. 

## 2. Results and Discussion

### 2.1. Analytical Method Validation of Indigotin and Indirubin: LOQ, LOD

#### 2.1.1. Importance of Chromatography

**Figure 2:**
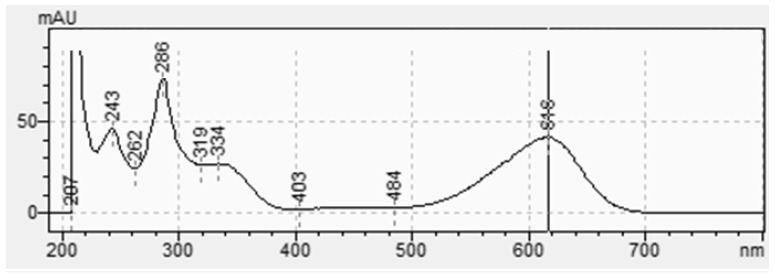
Absorption spectroscopy of indigotin with visible absorption maximum at 616 nm.

**Figure 3:**
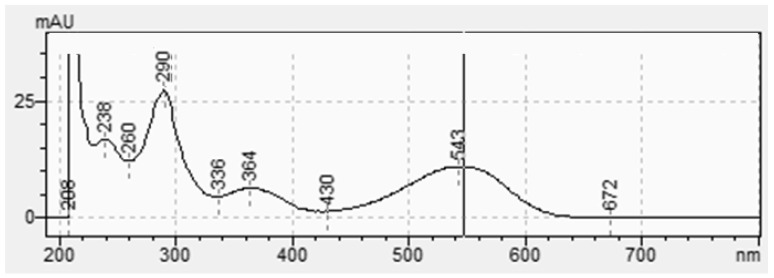
Absorption spectroscopy of indirubin with visible absorption maximum at 543 nm.

As shown in Figure 2 and Figure 3, indigotin and indirubin display a characteristic absorbance ranging from 500 nm to 700 nm and from 440 nm to 640 nm, respectively. They have different wavelengths at their maximum absorption, i.e., 616 nm (indigotin) and 543 nm (indirubin). Even if the two molecules have different wavelengths at their absorbance maxima, they overlap. This means that part of the absorbance measured at these maxima corresponds to the other compound. While indigotin is generally predominant, indirubin often contaminates samples. Consequently, it is crucial to specifically analyze for indigotin or indirubin; liquid chromatography with a UV–visible detector can solve this problem. Chromatographic analysis shows excellent separation of the molecules with retention times of 9.9 min and 15.4 min, respectively, for indigotin and indirubin.

#### 2.1.2. Analytical Method: Solubilization, Regression Coefficient, *LOD*, and *LOQ*

**Figure 4:**
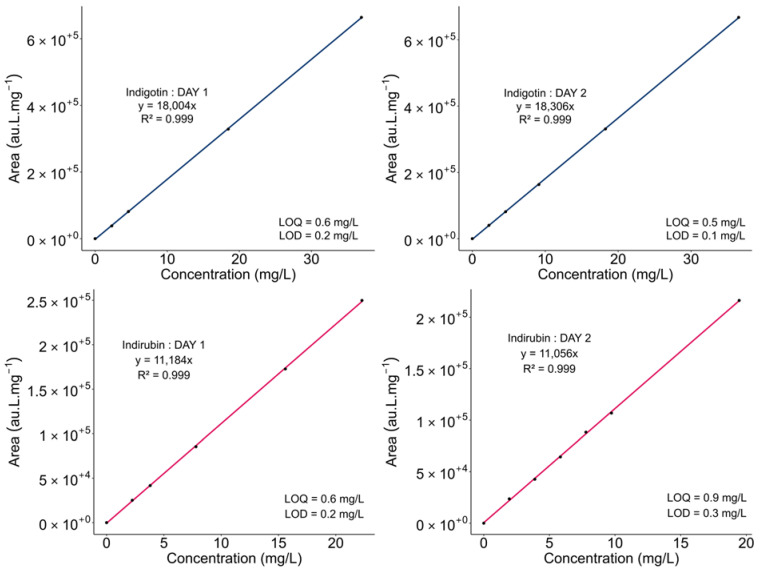
Calibration curves with analytic coefficients, regression coefficients, *LOD*, and *LOQ* of indigotin and indirubin on two successive days.

The objective was to identify a solvent able to dissolve indigoid compounds contained within pigments and to develop a HPLC method that can provide optimal separation between indigotin and indirubin. The main challenge is their low solubility in common solvents. A recent study showed promising results for the solubilization of natural indigo powder by comparing the following solvents: DMSO; acetone; ethyl acetate; methanol; and acetonitrile [24]. The best solubility was reported for DMSO. Other teams used ethyl acetate [21], organic and inorganic acids, or DCM [26] for the extraction of dye compounds, sometimes intended for HPLC analysis [27]. Other studies reported the use of *N*,*N*-dimethylformamide (DMF) [28] and *N*-methyl-2-pyrrolidone (NMP) [29] to dissolve indigotin and indirubin. Thus, an analogue of these solvents, DMAc (*N*,*N*-dimethylacetamide), was chosen due to its good solubilization properties, especially for indigotin (up to 36 mg/L in this study), and for its compatibility with chromatographic analyses. The HPLC method was developed from a comparative study on indigotin and indirubin in diverse natural indigos [30]. Different ranges of indigotin and indirubin were tested on two different days; the curves and data are depicted in the Figure 4, showing a good reproducibility and solution stability as the regression curve slopes are similar, i.e., 18,004 and 18,306 au.L.mg^−1^ for indigotin and 11,184 and 11,056 au.L.mg^−1^ for indirubin for days 1 and 2. The correlation was consistent in every case, with a regression coefficient greater than 0.999. The limits of detection (*LOD*) and quantification (*LOQ*) were determined. On day 1, the *LOD* for indigotin and indirubin was 0.2 mg/L and the *LOQ* was 0.6 mg/L. On day 2, the *LOD* was 0.1 mg/L for indigotin and 0.3 mg/L for indirubin. The *LOQ* was 0.5 mg/L for indigotin and 0.9 mg/L for indirubin. The ranges studied extended up to a maximum of 36.9 mg/L for indigotin and 22.3 mg/L for indirubin for the most evaluated ranges. For example, with DMSO, the solubility of indigotin was relatively lower, at 5 mg/L [31].

### 2.2. Comparison of Oxidation Techniques: Traditional Oxidation in Alkaline Medium, Oxidation with Iron (III) Chloride and Spontaneous Oxidation

#### 2.2.1. Traditional Oxidation in Alkaline Medium at pH 10

**Figure 5:**
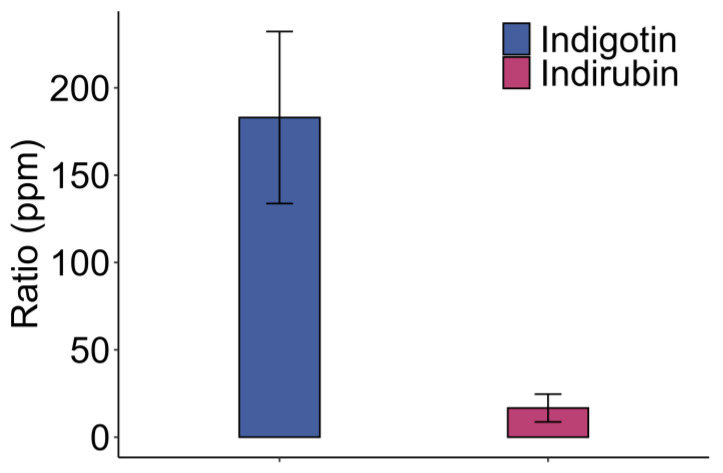
Oxidation with 30 min aeration at a pH of 10 from the summer batch.

Production of the pigment proceeds through several phases. First, the infusion of leaves in an aqueous medium at 60 °C allows for the release of indigoid precursors and free indoxyl molecules. Second, to produce indigotin, it is necessary to add a base for the easiest oxidation of indoxyl molecules. Thus, a 1 M sodium hydroxide solution was used to adjust the pH of the extract to 10 ± 0.2 [17]. This was followed by aeration of the extract medium for 30 min to provide the oxygen that promotes this oxidation (Figure 5). 

In the technical replication (similar extract), the free indoxyl present in the extract should be oxidized in the same way as indigotin and indirubin. However, in the traditional oxidation test, the indigotin levels from the technical replication did not follow this trend. The ratio of 129 ppm to 225 ppm describes a large standard deviation (Figure 5). These results show that air oxidation in an alkaline medium is not reproducible. Using this oxidation method, the results on the same extract showed ratios with a high standard deviation, despite using the same extract and conducting three technical replicates. To optimize the overall process for obtaining indigotin from woad, it is necessary to have a reproducible oxidation technique for the same extract, the technical replicates (different extracts from the same biological material) and for biological replicates (samples treated in the same way but using different biological materials). Two hypotheses are proposed to explain this variability. First, the current oxidation method is not reproducible and does not yield consistent indigotin ratios from the same extract (repeatability). Second, the batch of leaves exhibits biological variability, meaning that the initial quantities of indoxyl or precursors vary, which explains the differences between extracts (reproducibility). It was necessary to find an alternative way to oxidize the extract in order to observe the effects of this new approach on both repeatability and reproducibility. The oxidation technique must be rapid and necessarily reproducible. One study reported a ratio of 820 ppm based on an average of 17 different woad genotypes [32]. This study describes an indigotin equivalent using measured concentrations of isatan B and indican obtained by calculation. Although the value is higher, it does not take into account the formation of indirubin. Another study showed a ratio of 380 ± 120 ppm at 30 °C and 47 ± 12 ppm at 60 °C of infusion [33]. Although the results were lower for the 60 °C infusion, which seems inexplicable, the results at 30 °C followed the trend of the results described in Figure 5.

#### 2.2.2. Oxidation in Acidic Medium with Iron (III) Chloride

**Figure 6:**
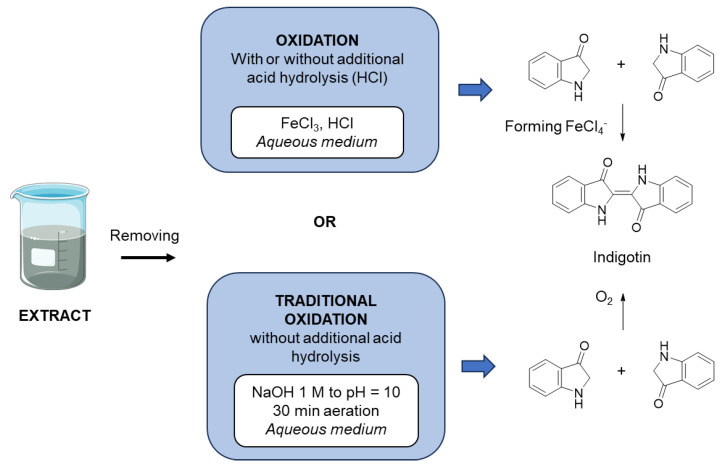
Oxidation methods used to obtain indigotin from woad extracts.

**Figure 7:**
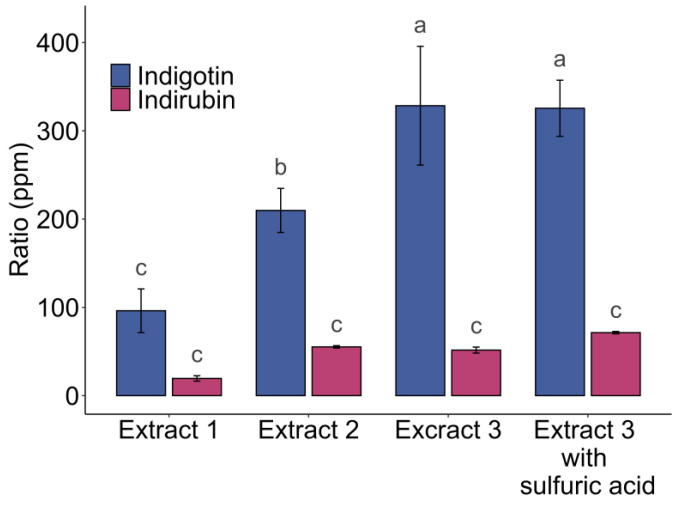
Oxidation by iron chloride and hydrolysis with hydrochloric acid on three different woad extracts and with sulfuric acid in Extract 3. Different letters above error bars (i.e., ±SD) indicate significant differences (*p* < 0.05) among groups (Tukey’s post-hoc test).

Various studies have been conducted on purple urine bag syndrome (PUBS), a benign urinary tract infection [25]. The symptoms of this infection are characterized by the violet-blue coloration of urine bags from catheters. This coloration is caused by the presence of indigotin and indirubin. This phenomenon results from the metabolism of tryptophan, which catabolizes into indole in the gastrointestinal tract, which is then converted into indoxyl sulfate in the liver. A research team investigated this infection and set up a method to easily measure the amount of indoxyl sulfate by indirect assay, forcing its conversion into indigotin. They used a Lewis acid, iron chloride (III), to oxidize indoxyl sulfate into indigotin. This protocol, initially used as a method to determine free indoxyl units, allows for the total oxidation of indoxyl into indigotin. A diagram outlining the protocol is shown in Figure 6. Three extractions were performed under the conditions described in Figure 7.

For each extraction (Extract 1, Extract 2, and Extract 3), three samples were taken and oxidized in the presence of FeCl_3_. An additional sample of Extract 3 was further hydrolyzed with H_2_SO_4_. The results of the indigotin and indirubin formation are presented in Figure 7. The indigotin ratios were 96 ± 25 ppm (Extract 1), 210 ± 25 ppm (Extract 2), and 326 ± 32 ppm (Extract 3). The indirubin ratios were 19 ± 3 ppm (Extract 1), 55 ± 1 ppm (Extract 2), and 78 ± 4 ppm (Extract 3). The oxidation method showed significant differences in the indigotin ratios depending on the extract’s hydrolyzation with H_2_SO_4_. For indirubin, the biological replicates were statistically similar. These significant differences demonstrated a technical difference due to the batch, which did not contain the same amount of precursors or indoxyl, as was observed with the use of alkaline oxidation. The standard deviation for the same extract increased with the increase in the indigotin ratio, which can be explained by the varying degrees of precursor hydrolysis into indoxyl among replicates of the same extract or an incomplete reaction. Indeed, at this concentration of hydrochloric acid, acid hydrolysis of glycosides occurs [34]. Therefore, an additional hydrolysis was performed on an extract to observe the incomplete hydrolysis of precursors with the use of hydrochloric acid alone, as shown in Figure 7.

The results showed significantly equivalent levels of indigotin, with or without the addition of sulfuric acid: 326 ± 32 ppm versus 328 ± 67 ppm, and 71 ± 1 ppm versus 52 ± 3 ppm for indirubin, for repetitions from Extract 3 (Figure 7). A halving of the standard deviation for indigotin was observed when using sulfuric acid, from 67 ppm to 32 ppm, while no differences were observed in indirubin levels. These observations suggest that there was a variation phenomenon within the same extract. This phenomenon can be caused by the partial hydrolysis of precursors for technical replications. The additional hydrolysis of indoxyl precursors induced by the addition of sulfuric acid can accelerate the hydrolysis step that hydrochloric acid partially achieves in a reaction time of 2 h, allowing for a more accurate assessment of the extract’s real potential to provide indigotin and indirubin, assuming that all precursors are hydrolyzed. 

In this context, the addition of an acidic hydrolysis is a technique used to determine the total capacity of the extract to produce indigotin and indirubin, aiming to complete the hydrolysis that has been partially achieved by endogenous enzymes. The issue of the reaction time was raised, particularly due to the necessity for achieving complete precursor hydrolysis when no additional hydrolysis is performed. In this context, the reaction time was increased to 24 h, allowing for both the total hydrolysis of precursors and complete oxidation of indoxyl molecules into indigotin/indirubin. In conclusion, this random variability in the batch, leading to variations in indigotin and indirubin ratios, initially stemmed from partial hydrolysis of the precursors. It is certain that there was a reaction between the precursors and certain compounds, both complete and random compounds in the batch, which was performed by the endogenous enzyme present in the leaves. This enzymatic hydrolysis may have occurred before, during, or after harvest, and/or not during infusion. The use of hydrochloric or sulfuric acid in this process is not feasible due to human and environmental toxicity. The challenge is ultimately to use enzymes capable of hydrolyzing these precursors using enzyme-assisted extraction (EAE). The disadvantage is that this step must be conducted after the infusion in order to not precipitate the indigotin with the leaves, which can then not be separated. Studies on these aspects are already underway, in particular, using β-glucosidase from various sources, some of which are bacterial [35,36,37].

#### 2.2.3. Spontaneous Oxidation of Free Indoxyl

**Figure 8:**
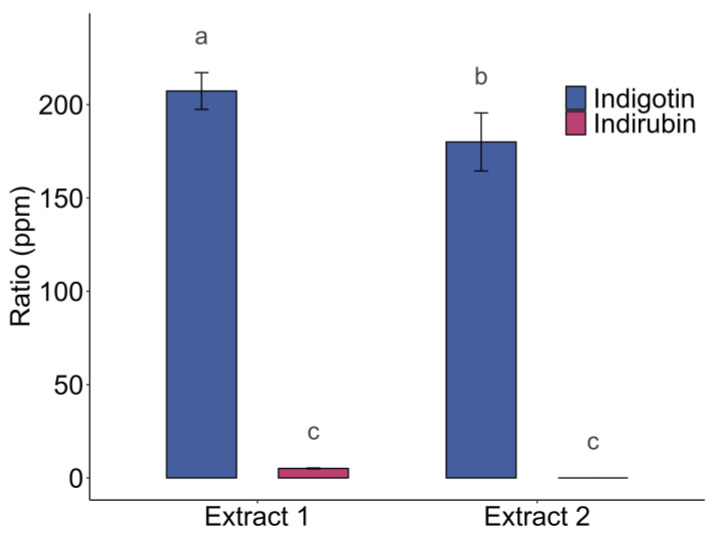
Spontaneous oxidation in two biologicals replications at pH 5 for 96 h in the dark at 25 °C. Different letters above error bars (i.e., ±SD) indicate significant differences (*p* < 0.05) among groups (Tukey’s post-hoc test).

Although oxidation in the presence of iron (III) chloride offers considerable advantages since it allows for indigotin to be obtained in high yields, it appears challenging to implement on a large scale, particularly due to its toxicity [38]. In this context, various studies on oxidation have shown that in the extract, free indoxyl oxidation occurs spontaneously, likely due to the oxygen present in the air (aerobic environment) [17,18,33]. One study demonstrated free indoxyl oxidation in two biological replicates (Figure 8). Indigotin was present at 207 ± 10 ppm (Extract 1) and 180 ± 16 ppm (Extract 2). Indirubin was negligibly present at 5 ± 0 ppm (Extract 1) and not detected in Extract 2. By leaving the extract at the same pH as during extraction (pH 5) for 96 h in the dark at 25 °C without stirring, spontaneous oxidation was observed within the medium. Indirubin occurs to a lesser extent under these conditions than during oxidation in an alkaline medium accompanied by aeration or with iron chloride (Figure 5 and Figure 7). These results suggest that the indigo pigment forms spontaneously without the addition of catalysts or basic agents, oxidizing the available free indoxyl. There are significant differences between biological replicates for the indigotin ratio (a, b) in Figure 8. that could have been caused by the non-homogeneity of indoxyl and precursor concentrations in the batch due to partial enzymatic hydrolysis by enzymes.

The composition of the indirubin-containing powders obtained using oxidation processes adapted for industrial scalability were analyzed and are compared in Table 1. The percentage of indigotin in the replicates using spontaneous oxidation is 19 to 21.6%, compared to 3.3 to 5.9% when using sodium hydroxide as the oxidation method. The percentage of indigotin was higher when using the spontaneous oxidation process, with the natural indigo obtained being purer and free of indirubin. When using sodium hydroxide, other compounds may precipitate. We could not analyze the powder obtained with iron (III) chloride as we directly analyzed the indigotin/indirubin content within the extract. Eight natural indigo powders obtained by an aeration process showed purities below 2% indigotin and below 0.5% indirubin, demonstrating lower values than those obtained in our results [30]. Using spontaneous dimerization, Jin and al. (2022) describe how Hurry (1930) found indigotin values ranging from 20 to 30%, showing the same trends as the results described in Table 1 [17,33].

**Figure 9:**
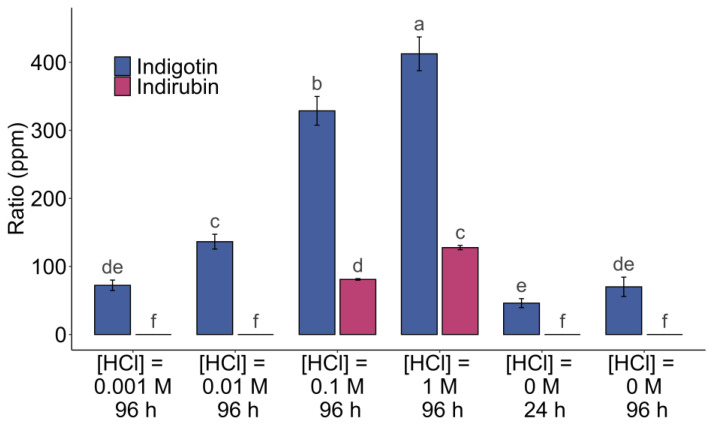
Spontaneous oxidation at different hydrochloric acid concentrations. Different letters above error bars (i.e., ±SD) indicate significant differences (*p* < 0.05) among groups (Tukey’s post-hoc test).

As shown in the previous sections, the extracts did not realize their full indigotin potential, as the precursors were not totally hydrolyzed. Therefore, we investigated the influence of additional hydrolysis using spontaneous oxidation as the oxidation method. These experiments showed an increase in the production of indigotin and indirubin with increasing HCl concentrations in the technical replication (Figure 9). The ratios of indigotin ranged from 72 ± 8 ppm to 412 ± 23 ppm, and from 0 to 128 ± 3 ppm for indirubin. Significant differences were observed among all trials when the acid concentration was increased by a factor of 10. A concentration of 0.001 M HCl did not seem sufficient to induce complete acid hydrolysis of the precursors. The recovered indigotin was thus produced from the oxidation of naturally hydrolyzed precursors through an enzymatic reaction. This was confirmed by the similarity in the results between this trial and spontaneous oxidation without the acid addition at 24 h and 96 h, indicating that a 24 h period is sufficient to oxidize indoxyl molecules into indigotin.

Furthermore, the presented results lead to several hypotheses. Indoxyl precursors did not seem to hydrolyze over time in the extract, indicating that there is no hydrolysis of precursors at a pH of 10 with sodium hydroxide, as suggested by Oberthür et al. [20]. Therefore, some non-hydrolyzed precursors can lead to indigotin through spontaneous oxidation in the extract without the addition of an oxidizing agent. The formation of indirubin seems to be favored in an acidic environment in combination with iron chloride, and also in basic media in combination with sodium hydroxide. 

### 2.3. Application of Acid Hydrolysis and Oxidation with Iron (III) Chloride to the Freezing Period Batch

**Figure 10:**
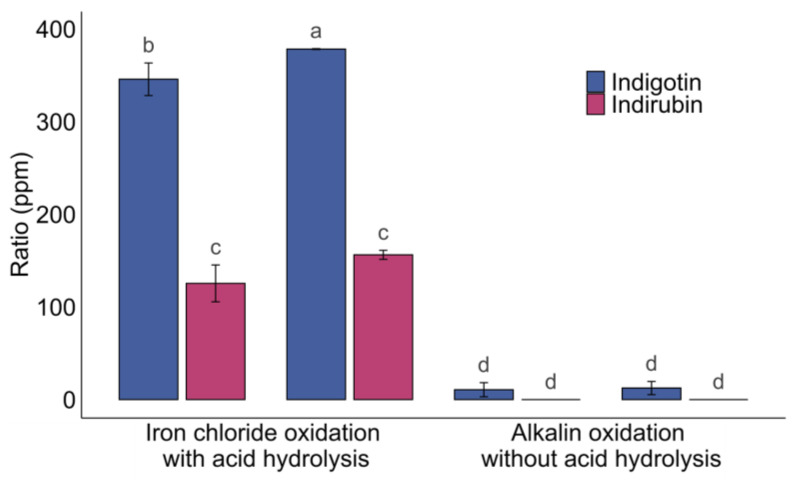
Comparison of iron chloride oxidation with acid hydrolysis and alkaline oxidation without acid hydrolysis on woad extract for the freezing period batch. Different letters above error bars (i.e., ±SD) indicate significant differences (*p* < 0.05) among groups (Tukey’s post-hoc test).

The summer batch shows interesting results in terms of indigotin amount isolated (129 ppm to 225 ppm), despite partial hydrolysis when using the current oxidation process in an alkaline medium (Figure 5). The results of the freezing period batch with the same process, on two biological replicates with three technical replicates per extract, show much lower indigotin levels (Figure 10). The indigotin ratios are 11 ± 8 ppm (extract 1) and 12 ± 7 ppm (extract 2). Indirubin is not detected in these extracts. Since these results are relatively weak, the hypothesis is that the precursors were not hydrolyzed. Thus, two extractions are performed on the freezing period batch and oxidation is carried with iron chloride as catalyst and hydrochloric acid for 24 h (Figure 10). The results showed indigotin ratios of 346 ± 23 ppm (extract 1) and 378 ± 30 ppm (extract 2), and indirubin ratios of 125 ± 20 ppm (extract 1) and 156 ± 5 ppm (extract 2). The results show a significant increase in indigotin and indirubin using iron chloride as oxidizing agent compared to alkaline oxidation (Figure 10). Comparing alkaline and acidic oxidation for the freezing period batch suggests that indoxyl precursors do not seem to be hydrolyzed into indoxyl prior to oxidation. Without indoxyl production, oxidation cannot occur directly on this extract. These results are in line with those obtained from the study of the hydrolysis of *O*-glycosides at different HCl concentrations, suggesting that there is no hydrolysis by NaOH at this concentration (Figure 9). Under FeCl_3_/HCl conditions, indigotin production during the freezing period follows the same trend as in summer, with maximum amounts of 402 ppm in the summer batch and 379 ppm in the freezing period batch. To our knowledge, this is the first time that an analysis of the evaluation of indigotin over a freezing period has been carried out. These results are interesting and may allow for the use of woad over a longer cultivation period. A study on *Phaius flavus* flowers showed a significant production of indigotin during freezing treatment [39]. These results may explain the indigotin ratios found in the extracts of the summer batch oxidized by spontaneous oxidation or by 30 min of aeration in alkaline medium (Figure 5 and Figure 8). Thus, hydrolysis and oxidation may have occurred after freezing the leaves. However, it does not explain why the batch of leaves freezing period culture show little indigotin despite the same treatment. These results indicate that the *O*-glucosides were hydrolyzed by the endogenous β-glucosidase, whereas no hydrolysis took place during the freezing period culture because the indoxyl molecules were not present (Figure 9 and Figure 10) [16]. It can be suggested that enzymatic material required for hydrolysis is either inactive or not produced during the freezing period culture.

## 3. Materials and Methods

### 3.1. Materials and Consumables

All reagents used had a minimum purity of 95%, unless otherwise specified. The standards used for HPLC analysis included synthetic indigotin with 95% purity from Sigma-Aldrich and indirubin (≥95%) Phyproof^®^ from PhytoLab (Vestenbergsgreuth, Germany). Anhydrous iron (III) chloride (FeCl_3_) and sodium hydroxide 99% were purchased from Laboratoire Verbiese (Merville, Nord, France). *N*,*N*-Dimethylacetamide 99,5% (DMAc) was purchased from Acros Organics at Germany and was taken from Carlo Erba Reagents. For the HPLC analysis, HPLC-grade Methanol (MeOH) was purchased from Carlo Erba Reagents and formic acid was purchased from Honeywell Fluka™. Hydrochloric acid 37% and sulfuric acid was obtained from Sordalab (Étampes, France). The plants were infused in water and the oxidation was performed on a Macherey-Nagel Nanocolor^®^ VARIO 3 heating block with 10 mL Pyrex^®^ glass test tube. Solvent evaporation was carried out with a greenVac PC3001 Vario rotary evaporator (Vacuubrand, Wertheim, Germany). Centrifugations were carried out with a SIGMA^®^ 3k15 refrigerated centrifuge. 

### 3.2. HPLC Analyses

HPLC analyses were carried out using an LC-20ADSP pump (Shimadzu, Marne la Vallée, France) equipped with an Uptisphère UP5HDO-250/046 column (Interchim, Montluçon, France) (250 mm × 4.6 mm, 5 µm) maintained at 25 °C and a diode array detector (DAD) SPD M20A. Methanol–water (0.1% formic acid) 3:1 (*v*/*v*) was used as an isocratic mobile phase (flow rate 1.0 mL/min) for 18 min. The retention times were 9.9 min for indigotin and 15.4 min for indirubin at wavelengths of 616 nm and 543 nm, respectively.

### 3.3. Plant Material and Growth Condition

**Figure 11:**
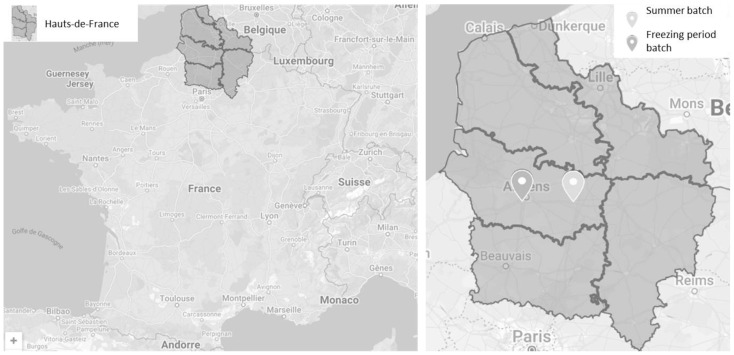
Map of France and Hauts-de-France with landmarks used by the field (map created with MyMaps).

Two batches of crops were grown, one in summer and one in a freezing period. The cultivation site for the summer batch is located in Soyécourt (Latitude 49.86261, Longitude 2.79524) and is owned by Company SAS Couleurs Végétales de France, as shown in Figure 11. The cultivation site for the freezing period batch is located in Salouël (Latitude 49.87090, Longitude 2.24173) and is owned by Company Savonnerie des Hauts-de-France. In both cases, the plants were cultivated in open fields from seeds sown in April. The harvesting for the summer batch took place in July, while the harvesting for the freezing period batch occurred in November, during the freezing period. The harvested samples were stored in a freezer at −32 °C until extraction.

### 3.4. Aqueous Extraction 

From 1 g to 5 g of uncrushed leaves were added to tap water at a ratio of 1:5 *w*/*v* (1 g of biomass: 5 mL of tap water, pH 7 ± 0.2) previously heated to 60 °C. The infusion was carried out at 60 °C in the dark without stirring for 30 min. The leaves were removed from the extract with pliers and the supernatant was collected and filtered on paper with a porosity of 10–20 µm under vacuum to remove any remaining plant residues. The extracts studied were obtained from the summer batch. Only Section 2.3. refers to the freezing period batch. The bar plots shown are obtained from the biological replicate, except for the hydrolysis with oxidation by dimerization study in Figure 9, Section 2.2.3, where tests were conducted on the same extract (technical replicate). Each technical repetition was performed in two or three replicates.

### 3.5. Alcaline/Air-Mediated Oxidation

The pH of the extract was adjusted to 10 ± 0.2 with a 1 M NaOH solution to promote indigotin formation. The medium was then aerated for 30 min with compressed air through a flexible silicone tube with an internal diameter of 4.5 mm. 

### 3.6. Oxidation in the Presence of FeCl_3_ in Aqueous Acidic Medium

A total of 500 µL of the extract was transferred into a 10 mL pyrex tube and 280 µL of 1 mM FeCl_3_ solution in 1 M aqueous HCl or 1 mM FeCl_3_ solution in CH_2_Cl_2_ were added. If further hydrolysis was required, 56 µL of 50% sulfuric acid was added beforehand. The medium was left at 70 °C for 2 h or 24 h using a Macherey-Nagel Nanocolor^®^ VARIO 3 heating block. Samples were stored at −20 °C until HPLC analysis.

### 3.7. Spontaneous Oxidation

The entire extract was left at 25 °C for 96 h in an oven in the dark. A spontaneous oxidation test with additional hydrolysis was carried out on 1 mL of extract at different concentrations of acid. This hydrolysis occurred at a pH of 1 M, 0.1 M, 0.01 M, and 0.001 M; each test was carried out in triplicate. A total of 90 µL of acid hydrolysis was carried out using HCl at an appropriate concentration and was left for 96 h at 25 °C in an oven. A total of 90 µL at the same concentration of NaOH was added to the medium 24 h before the end of the reaction in order to neutralize it and maintain similar conditions to the oxidation without a pH adjustment. Samples were stored at −20 °C until HPLC analysis.

### 3.8. Treatment before HPLC Analyses

For the samples oxidized with alkaline/air-mediated and spontaneous dimerization, the extract containing the precipitate was centrifugated at 5000 rpm, 4 °C, using the SIGMA^®^ 3k15 refrigerated centrifuge. The recovered residue was then washed once with distilled water to remove any remaining traces of extract and was dried in an oven at 40 °C for 48 h. A total of 1 mg of the dry solid was solubilized into 5 mL of DMAc prior to HPLC analysis. For the samples oxidized in the presence of FeCl_3_ with acidic hydrolysis, 1 to 5 mL of DMAc were added to the medium. The samples were then filtered with 0.2 µm PTFE filters and 5 µL of this solution was injected and analyzed by HPLC-DAD.

### 3.9. Ratio and Purity Calculation

The ratio represents the amount of indigotin or indirubin per mass of fresh leaf in ppm or µg/g and was calculated by the following Formula (1):

(1) $$Ratio=\frac{w1}{w2}$$

where the weight 1 (*w*1) is the weight of indigotin calculated according to HPLC results in µg and weight 2 (*w*2) is the weight of the fresh leaf in g.

The purity (%) represents the amount of indigotin or indirubin contained per mass of indigo powder in the percentage (%) and was calculated by the following Formula (2):

(2) $$\%=\frac{w1}{w3}$$

where weight 1 (*w*1) is the weight of indigotin or indirubin calculated according HPLC results in µg and weight 3 (*w*3) is the weight of indigo powder in mg.

### 3.10. Statistical Analyses

The limit of detection (*LOD*) (3) and limit of quantification (*LOQ*) (4) were calculated using the following formula:

(3) $$LOD=3.3\times \frac{\sigma }{a}$$

(4) $$LOQ=10\times \frac{\sigma }{a}$$

where *σ* is the standard error of intercept (the standard deviation around the regression line for all the data) and a is the slope of the linear regression.

RStudio software (RStudio 2024.04.1+748 “Chocolate Cosmos” Release for windows) was used to construct the bar plots and to display the statistical differences. The following packages were used to construct the bar graphs and plot the statistical differences: datasets; multcompView; dplyr; rstatix; readxl; corrplot; tidyr; tidyverse; digitize; ggpubr; and forcats. The variance of the data was analyzed using ANOVA. Data were analyzed with the Tukey test, and significant differences were represented by letters on the figures. The upper and lower error bars on the bar plots represent two standard deviations (2SD). 

## 4. Conclusions

In this work, HPLC-DAD analysis was highlighted as an essential tool with which to identify and quantify indigotin and indirubin from natural indigo obtained from woad (*Isatis tinctoria* L.). This analytical method was described with a linear regression up to 36.9 mg/L and 22.3 mg/L for indigotin and indirubin, respectively, and a good resolution. Thanks to this precise analytical method, two alternative oxidation methods have been compared to the traditional alkaline oxidation [17]. First, the iron (III) chloride method quickly forms indigotin and requires an acidic medium. Second, spontaneous oxidation does not need any extra reagent, but needs more time to be completed; the oxidation is slower than using iron chloride or a traditional air-mediated alkaline medium. However, spontaneous oxidation showed a more interesting indigotin content than the current alkaline oxidation process, leading to a powder containing 21.6% of indigotin compared to 5.9%, without the detectable formation of indirubin. 

These different processes highlighted the presumed origin of the non-repeatability of indigotin ratios obtained between extracts from the same batch of leaves and the biological variability resulting from the partial hydrolysis of glycosylated indoxyl precursors. This phenomenon was especially illustrated by the use of hydrochloric acid during oxidation with FeCl_3_ in an acidic medium. This issue is exacerbated during freezing periods. The results showed that these precursors were not hydrolyzed; thus, indoxyl was not formed and therefore there was no oxidation to indigotine. This suggests a lack of enzymatic degradation during freezing periods. Once these precursors were hydrolyzed in acidic conditions, ratios of indigotin equivalent to those of the summer period were recovered. These results are promising to consider when woad harvesting throughout the year. Depending on agricultural difficulties during the freezing period, with a modified existing process, incorporating an acidic reagent or enzymes can achieve the complete hydrolysis of precursors.

## Figures and Tables

**Table 1:** Difference in purity obtained for indigotin and indirubin between the two types of oxidation.

Pigment Produced by Spontaneous Oxidation (Figure 8, Extract 1)		Pigment Produced by Alkaline Oxidation (Figure 5)	
% Indigotin	% Indirubin	% Indigotin	% Indirubin
19.0	0.2	3.3	0.4
21.6	0.1	5.9	0.6
20.4	0.4	5.1	0.2

## Data Availability

Data are contained within the article.
